# Modelling behavioural intention to buy apartments in Bangladesh: An extended theory of planned behaviour (TPB)

**DOI:** 10.1016/j.heliyon.2022.e10519

**Published:** 2022-09-06

**Authors:** Muhammad Ariful Islam, Zainil Hanim Saidin, Meor Azli Ayub, Md Shamimul Islam

**Affiliations:** a School of Distance Education, Universiti Sains Malaysia, Penang, Malaysia; b Brac Business School, Brac University, Dhaka, Bangladesh

**Keywords:** Real estate, Exploratory factor analysis, Extended TPB, Behavioural intention, Measurement items, Theoretical model

## Abstract

Understanding the elements that influence buyers' purchase intentions is critical for real estate companies. The goal of this study is to develop a model for investigating behavioural intentions to purchase apartments in Bangladesh. To obtain the preliminary elements, the Theory of Planned Behaviour (TPB) was used as the underpinning theoretical framework. Other elements relevant to the study context were identified by an extensive literature review, which was subsequently evaluated by industry experts. A systematic questionnaire was used to collect two hundred and thirty-six samples. To find the relevant factors, an exploratory factor analysis (EFA) was used. In addition to the criteria mentioned by the TPB, the results reveal three additional aspects: perceived physical quality, access to money, and favourable government policy. The study contributes to the literature by presenting an extended TPB model suitable for studying behavioural intention to buy apartments in an emerging country.

## Teaser

Real estate; Exploratory eactor analysis; Extended TPB; Behavioural intention; Measurement items, Theoretical model.

## 1 Introduction

In the investment landscape, real estate is a significant and important asset class (Ghent et al., 2019). It is critical for both buyers and the housing industry to investigate the elements that influence buyers' decision-making in real estate markets. To achieve a competitive advantage, real estate companies must understand consumer behaviour while purchasing apartments. It is one of the most important economic decisions that people make, and making well-informed decisions necessitates gathering a lot of data (Haddad et al., 2011; Zadkarim and Emari, 2011). As a result, it’s vital to comprehend the motivations of potential apartment buyers.

Many behavioural theories in the field of psychology have been created around the world that may be used in marketing and relevant to real estate (DeLisle, 2012). The majority of previous studies used TPB to analyse consumer buying behaviour in the real estate environment. For instance, Kamal and Pramanik, 2015a, Kamal and Pramanik, 2015b used the TPB as their research model to investigate customers' intentions to buy apartments in Dhaka, Bangladesh. The same hypothesis was used in another study by Judge et al. (2019) to predict buyer intent to purchase sustainable housing in Australia. AL-Nahdi et al., 2015a, AL-Nahdi et al., 2015b, Al-Nahdi et al., 2015c, AL-Nahdi et al., 2015d utilized TPB to estimate apartment buying intentions in Saudi Arabia. Other significant parameters besides TPB were not considered in these three previous studies (Kamal and Pramanik, 2015a, Kamal and Pramanik, 2015b, 2015b; AL-Nahdi et al., 2015a, AL-Nahdi et al., 2015b, Al-Nahdi et al., 2015c, AL-Nahdi et al., 2015d; Judge et al., 2019).

Ajzen (1988) and Ajzen (1991) proposed TPB to study behavioural intention. Some critics, however, claim that the theory ignores important factors that determine behavioural intention (Yazdanpanah and Forouzani, 2015). As a result, TPB cannot be utilised only to investigate apartment-buying intentions. In the lack of a proper measuring structure in the current setting, it is critical to broaden TPB to include other contextual elements. As a result, the goal of this study is to develop a model for analysing the behavioural intention of buying an apartment in Bangladesh. This study addresses the following research questions. Q1. What are the factors affecting the behavioural intention of consumers to buy apartments? Q2. How to identify the relevant factors and propose a model in the given context? TPB has been adopted as an underpinning theoretical framework. Additional criteria were uncovered during the literature review, which was later confirmed by industry experts. An exploratory factor analysis (EFA) was done to identify the relevant factors. Aside from TPB, the findings point to three more factors. Finally, a model is proposed based on the findings.

This study adds to the body of knowledge in two ways. To begin, this research presents a model for investigating behavioural intentions in apartment purchasing. There are few studies in the literature that investigate buyer behaviour in the context of a real estate market in an emerging economy. Second, this study expands on the TPB theory by incorporating three new variables: perceived physical quality, access to money, and government favourable policy. The extended theory is appropriate for researching behavioural intentions to buy apartments in developing countries.

This paper is divided into six sections. Following the introduction in section 1, section 2 discusses the theoretical background and conceptualization of the attributes, subjective norms, perceived behavioural control, perceived physical quality, access to money, government policy, intention, and consumer behaviour. Section 3 then provides a detailed discussion of the methods used. Section 4 presents the findings and discussion, while Section 5 presents the measurement items and theoretical implications. Finally, section 6 discusses the conclusion, limitations, and future research suggestions.

## 2 Literature review

This section discusses the theoretical background, followed by operationalizing the variables that are found relevant for the proposed model.

### 2.1 The theoretical background

The theory of planned behaviour was proposed as an extension of the theory of reasoned action (Ajzen, 1988, 1991). According to the TPB, consumer behaviour can be predicted by the consumers' attitudes toward the behaviour, subjective norms regarding the behaviour, and perceived control over performing the behaviour (Ajzen, 1991; Sheeran et al., 2003). It is the subject of considerable attention in relation to beliefs. Ajzen (1991) emphasised three types of beliefs related to the three predictors of intention: behavioural beliefs, which are assumed to influence attitudes toward the behaviour, normative beliefs, which serve as the underlying determinants of subjective norms, and control beliefs, which serve as the foundation for perceptions of behavioural control. The degree to which a person has a favourable or unfavourable opinion of the behaviour is referred to as consumer attitudes. The perceived social pressure to perform or not execute the behaviour is referred to as subjective norms. Perceived behavioural control relates to the perceived ease or difficulty of carrying out the behaviour, and is thought to be influenced by expected obstacles (Ajzen, 1991; Sheeran et al., 2003). Despite its dominance in the study of human behaviour, recent criticism alleges that it was impractical in a longitudinal study (Sniehotta et al., 2014). Furthermore, many significant elements that have been discovered to be important determinant factors influencing human behaviour were missed by this theory (Yazdanpanah and Forouzani, 2015). As a result, TPB expansion is required.

Additionally, the behavioural economics theory suggests that human behaviour differs from the standard behavioural models in reality and it mostly matters in economic reality e. g. finance and savings (Mullainathan and Thaler, 2000). The foundation of behavioural economics is based on psychology and economics. This theory primarily investigates biases, tendencies and heuristics behaviours of people while making economic decision (buying decision for example) (Thaler, 1980). This study incorporates finance, perceived physical quality and government favourable policies/incentives to study the behavioural intention to buy the apartment. These three factors are related to economic benefit and can be perceived as savings. Therefore, behavioural economics theory also explain the relationship of variables of interest in our model. As a result, the goal of this study is to investigate the variables determining behavioural intention to purchase an apartment. The variables of TPB and the other three variables identified as important in the exploratory factor analysis (EFA) study are discussed in the following sections.

### 2.2 Attitude

Attitude is defined as a psychological inclination communicated by evaluating a specific substance with some level of approval or disapproval (Ajzen, 1988). The attitude of a person toward a particular activity is referred to as their liking or dislike of that activity. Customers' purchasing attitudes have a significant impact on their purchasing intentions (Kamal and Pramanik, 2015a, Kamal and Pramanik, 2015b, 2015b). A favourable attitude toward purchasing an apartment is a strong predictor of future intentions (Judge et al., 2019).

**Figure 1:**
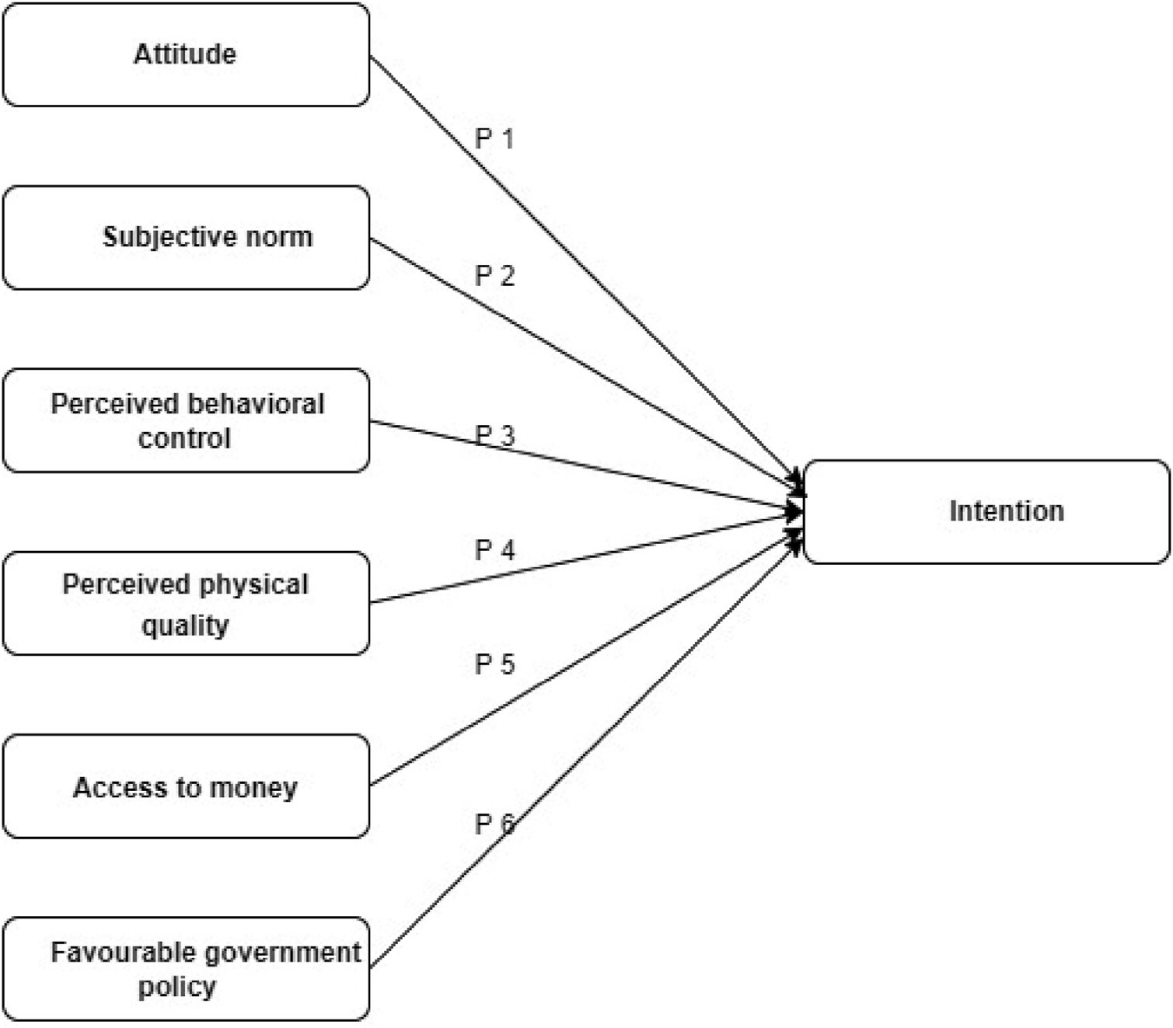
The proposed extended TPB Model in the real estate context.

According to Kamal et al. (2016), there is a significant relationship between attitude and apartment purchasing intentions in Bangladesh, which is supported by Saudi residents looking to buy real estate (AL-Nahdi et al., 2015a, AL-Nahdi et al., 2015b, Al-Nahdi et al., 2015c, AL-Nahdi et al., 2015d). Wibawa, Hartoyo, and Hartoyo (2017) and Jain (2020) found that attitude has a positive and significant effect on apartment purchase intentions. Other studies have discovered that attitudes have a positive influence on behavioural intentions to buy an apartment (Yazdanpanah and Forouzani, 2015; Khoo et al., 2019). Based on previous research findings, it is proposed that attitudes have a positive effect on the intention to purchase apartments (Figure 1: P1).

### 2.3 Subjective norm

Subjective norm is the outcome of pressure placed on a person to do or refrain from performing a behaviour (Han and Kim, 2010; Ajzen, 1991). Individuals' ideas about performing or not performing specific behaviours, as well as motivation and willingness to do or not do something thought significant, are referred to as subjective norms (Utami, 2017). It denotes the societal pressure to engage in or refrain from a behaviour (Bai et al., 2019). Customers' intentions to buy a house from the real estate sector were influenced by subjective norms in the case of real estate purchasers (AL-Nahdi et al., 2015a, AL-Nahdi et al., 2015b, Al-Nahdi et al., 2015c, AL-Nahdi et al., 2015d).

Subjective norms have a favourable impact on behavioural intention (Yazdanpanah and Forouzani, 2015). Jain (2020) discovered that subjective norm is positively associated with luxury purchasing intention in another study. It was also discovered that the positive influence of subjective norms resulted in stronger intentions to purchase real estate (AL-Nahdi et al., 2015a, AL-Nahdi et al., 2015b, Al-Nahdi et al., 2015c, AL-Nahdi et al., 2015d). Furthermore, numerous research has demonstrated that subjective norms have a strong positive influence on the intentions to purchase an apartment (Wibawa et al. 2017; Judge et al., 2019). Hence, this study proposed that subjective norm has a positive impact on the intention to buy apartments (Figure 1: P2).

### 2.4 Perceived behavioural control

Perceived behavioural control is a measurement of an individual’s opportunity and ability to do a behaviour when they believe they have the power to act or decide with that behaviour (Mamun, 2018). Perceived behavioural control, according to Ajzen (2015), can prevent individuals from executing a behaviour or make it easier for them to perform a behaviour when obstacles or resources are present. Perceived behaviour control has been established as a predictor of house-buying intention by researchers (Judge et al., 2019). It is also strongly linked to one’s beliefs about the influence of both situational and internal factors on one’s ability to accomplish a behavioural (Tan et al., 2017).

Wibawa et al. (2017) discovered that having easy access to information, as well as adequate money and time, is important when purchasing an apartment from the real estate sector. Numerous studies have found a positive relationship between perceived behavioural control and purchase behaviour intention (Yazdanpanah and Forouzani, 2015; Wibawa et al. 2017; Judge et al., 2019). Other studies have also confirmed the positive relationship between perceived behavioural control and consumer purchasing intentions in the real estate market (Khoo et al., 2019). As a result of these studies, it was proposed that perceived behavioural control has a positive effect on the intention to purchase apartments (Figure 1: P3).

### 2.5 Perceived physical quality

Customers' purchasing decisions for residential property are heavily influenced by quality (Rachmawati et al., 2019). When purchasing an apartment, the quality of the apartment, particularly the parking facility, the environment, proper ventilation, interior design, utility services, fittings, and construction quality, are all critical considerations (Kamal and Pramanik, 2015a, Kamal and Pramanik, 2015b, 2015b). According to Sonia (2020), project quality, construction quality, and environmental quality all influence the decision to acquire an apartment in Dhaka.

House features include house design, building quality, interior and exterior designs, and finishing. These features are expected to influence an individual’s house purchase decision (Chia et al., 2016). The quality of the construction, ventilation, parking, apartment design, and water supply are all important factors to consider when making a purchase (Kumar and Khandelwal, 2018). For the living quality of an apartment, an excellent water supply and drainage system are also essential (Kamal and Pramanik, 2015a, Kamal and Pramanik, 2015b, 2015b). Customer intent to acquire residential property is heavily influenced by quality (Kamal and Pramanik, 2015a, Kamal and Pramanik, 2015b, 2015b; Maoludyo and Aprianingsih, 2015; Rachmawati et al., 2019; Khoo et al., 2019). In light of past research, it is hypothesised that perceived physical quality has a beneficial impact on the desire to purchase an apartment (Figure 1: P4).

### 2.6 Access to money

When it comes to purchasing a home, money is a major consideration for customers (Khare and Kader, 2016). Purchasers of real estate must borrow substantial sums of money and pay interest on their loans. This price premium is used to determine whether or not a customer is willing to buy a product (Numraktrakul et al., 2012). If middle-income people had easier access to housing finance, they will be more inclined to buy apartments. The demand for home finance in Bangladesh was BDT 1496 billion in 2019, according to Bangladesh Bank, and it is constantly increasing (Sonia, 2020).

Financial factors had the largest influence on purchasing decisions, according to Paço and Raposo (2009). Finance has always been seen as a critical aspect of the real estate industry’s development. Customers are more interested in buying apartments and houses if they can get long-term, low-cost financing (Sonia, 2020). Previous studies have discovered a positive connection between financial resources and the intent to purchase an apartment (AL-Nahdi, 2015; Chia et al., 2016; Khoo et al., 2019). As a result, it is suggested that having access to money has a beneficial impact on the intention to own an apartment (Figure 1: P5).

### 2.7 Favourable government policy

An enabling policy makes it easier for the market to produce and distribute housing while also ensuring that it is done efficiently and equitably (Chowdhury, 2013). Governments can also have an impact on the construction industry as investors or users of buildings (Matisoff et al., 2016). Differentiated price and trade restriction policies for housing, as well as government monetary subsidies, can encourage developers to build affordable housing (Zhang et al., 2018).

Financial incentives, easy loans, tax benefits, and subsidies can all help to attract more potential home buyers (Ghodrati et al., 2012). Previous research has found that government policies such as incentives have a significant positive effect on apartment purchases (Matisoff et al. 2016; Glaeser et al., 2017; Alam, 2018). As a result, it is proposed that favourable government policy has a positive effect on the intention to buy apartments (Figure 1: P6).

### 2.8 Intention

In general, a person’s intentions are influenced by a variety of personal and influential beliefs. The dependent variable is purchase intention, which is predicted by an independent variable such as attitude, perceived behaviour control, and subjective norm (Ajzen, 1991, 2015). Customer purchase intention examines the reasons why customers choose and buy a particular brand, as well as the customers' preferences when purchasing a product or service (Hoe et al., 2018). When participants resolve to achieve their goals, they will have a reason to do so. No further action can be taken unless that motive is present (Mamun, 2018).

Purchase intention influences consumer behaviour positively (Arora and Sahney, 2018). Intention denotes a person’s desire to perform the behaviour, and it is a direct antecedent of behaviour (AL-Nahdi et al., 2015a, AL-Nahdi et al., 2015b, Al-Nahdi et al., 2015c, AL-Nahdi et al., 2015d). In the case of apartment purchases, the purpose comes before the purchase behaviour (Numraktrakul et al., 2012).

### 2.9 Consumer behaviour

Understanding consumer purchasing behaviour has become a critical issue in recent decades (Singh et al., 2018). Consumer behaviour is the study of individuals, groups, or organizations and the processes they use to select, secure, use, and dispose of products, services, experiences, or ideas to satisfy needs and the impacts that these processes have on the consumer and society (Stankevich, 2017). According to Dudovskiy (2015), consumers purchase behaviour is the result of an individual’s necessities and desires, and purchases are made to fulfil these desires. Consumer behaviour refers to the process that consumers go through when making purchases, and it includes factors that influence their decisions (Stankevich, 2017). So, consumer behaviour encompasses more than just making a purchasing decision or the act of purchasing; it also encompasses consumer interaction and the wide range of experiences associated with consuming (Nolcheska, 2017). Consumer behaviour is concerned with how individual consumers and families or households decide to spend their available resources (time, money, and effort) on consumption-related items (Karim, 2020). Previous research has predicted that intention influences consumer behaviour (AL-Nahdi et al., 2015a, AL-Nahdi et al., 2015b, Al-Nahdi et al., 2015c, AL-Nahdi et al., 2015d). Thus, studying both intention and consumer behaviour at the same time is illogical and necessitates a longitudinal study. As a result, this study assumes that intention will reflect behaviour.

## 3 Methodology

### 3.1 The proposed model

The proposed model is depicted in Figure 1. The model demonstrates that six factors influence behavioural intention, which leads to actual behaviour, such as purchasing an apartment. Attitudes, subjective norms, and perceived behavioural control obtained from TPB and EFA suggested perceived physical quality, access to money, and favourable government policy. Consumer behavioural intention is a very complex study that cannot be fully explained by a single model or theory. According to the proposed model, six variables can predict intention and assumed intention will reflect actual behaviour. This model adds to the literature on the behavioural intention to buy apartments. The model expanded on the TPB model by incorporating three additional latent variables: perceived physical quality, access to money, and favourable government policy, all of which are important in apartment purchasing.

### 3.2 The case background

Dhaka, the capital of Bangladesh, is one of the most densely populated areas in the world, with a density of 23,234 people per square kilometre (Awwal, 2019). Bangladesh’s public sector is struggling to meet the housing needs of Dhaka’s growing population. Over the last four decades, the private real estate sector has played a significant role in apartment supply. Their annual contribution is approximately 15,000 apartments (Seraj, 2012). People are interested in buying apartments in mid or high-rise buildings from the private real-estate market because there is a scarcity of buildable land and land values are high (Mohiuddin, 2014). Family patterns have evolved, and the traditional joint family is no longer the norm (Samad, 2015). The traditional family pattern has given way to a single-family pattern, which has increased the demand for Dhaka apartments (Seraj, 2015). In most cases, an apartment building houses a nuclear family of a husband, wife, and children (Ahmad, 2019).

In Bangladesh, a lack of defined income level makes it difficult to identify targeted clients (Chowdhury, 2013). Due to this, the appropriate product is not being developed to meet the needs of city people' consumer behaviour, leading to unsold or ready-to-move-in flats. Therefore, research into what factors influence people’s intentions to buy apartments in Bangladesh is necessary. As a result, it is critical to develop a model for studying customer behaviour. Besides adopting the variables from the TPB, this study also identified other variables from the literature review. The following variables have been identified including, project facilities (Kamal and Pramanik, 2015a, Kamal and Pramanik, 2015b, 2015b), environmental condition (Chia et al., 2016), location facility (Kumar and Khandelwal, 2018), perceived physical quality (Jamil et al., 2018), promotion (Rachmawati et al., 2019), price (Sonia, 2020), government incentive, policy and regulation (Glaeser et al., 2017; Alam, 2018) and finance (Khoo et al., 2019). To establish the key elements, an EFA study was recommended. The EFA was conducted using a structured questionnaire approach (Appendix A). It was then evaluated through consultation with experts in the relevant industry, including buyers, managers, and researchers. The EFA, as well as the data gathering process, are discussed in the following sections.

### 3.3 Sampling design

This study’s sample population consists of people seeking an apartment in Dhaka. We interacted with real estate salespeople to develop a list of possible Dhaka apartment buyers. Similarly, the Real Estate and Housing Association of Bangladesh (REHAB) hosts an annual housing show to collect information from visitors interested in buying an apartment. We received names of possible purchasers from REHAB and real estate professionals. According to the REHAB, this real estate market has 40,000–50,000 potential buyers. However, no source defines the actual number of people searching for an apartment on the real estate market. As a result, no sampling frame exists. Therefore, this study used a non-probability sampling method in the absence of a sampling frame.

In terms of sample size, the majority of prior studies used Gpower software to calculate the minimal sample size with a predictive power of 0.95 (Mahmud et al., 2017). According to the calculations, a minimum sample size of 146 is needed with six predictors (moderate effect size is 0.15). This study obtained 251 responses out of 400 self-administered questionnaires handed out. A total of 251 questionnaires were returned, with 236 of them including complete responses. As a result, this study relied on cross-sectional data and that is achieved sufficient samples for EFA study.

To fulfil the study’s objectives, certain sequential steps were taken to collect data. The questionnaire was distributed to the respondents through an online Google form. The questionnaire requests information on project facilities, environmental conditions, location facilities, perceived physical quality, promotion, price, government incentive policy and regulation, attitude, subjective norms, perceived behaviour control, and finance. The entire data collecting process took three months, from April 2020 to June 2020. The study also employed the reminder technique to accelerate the response time and response rate through a phone call. The informed consent was obtained from all participants in the research.

### 3.4 Data analysis method

#### 3.4.1 Exploratory factor analysis (EFA)

Exploratory factor analysis (EFA) is a data-driven approach to factor analysis and is used to extract a smaller number of common factors that represent or explain the common variance of a larger set of manifest variables (Watkins, 2018). Factor analysis is a widely used technique for identifying latent constructs underlying questionnaire responses (Steiner and Grieder, 2020). According to Brown (2015), "a factor is an unobservable variable that influences more than one observed measure and accounts for the correlations between these observed measures." In other words, the observed measures are linked because they are caused by the same factor. EFA describes how the study was carried out and presents the findings in sufficient detail, clarity, and coherence to support the validity of the findings and justify the study’s conclusions (Appelbaum et al., 2018). EFA is a multivariate statistical method that has become an important tool in the development and validation of psychological theories and measurements (Watkins, 2018).

This study has undertaken an EFA approach since there is a lack of an established measurement scale to study consumer behavioural intention in the real estate industry, particularly with respect to buying an apartment. This study has developed the initial questionnaire based on the literature review that has been subsequently consulted with and validated by the industry experts. The initial questionnaire contains a total of 63 questions, and after the EFA, the final questionnaire (see Appendix A) has been designed with 35 items.

## 4 Results and discussion

### 4.1 Descriptive statistics

Table 1 represents the demographic analysis of the respondents. Out of the 236 respondents, there are 203 (86%) male and 33 (14%) female respondents. In Bangladesh, male are the main earning person, so maximum respondents' interested purchasing apartment are male. Most of the respondents (97.9%) are married in the survey. In Bangladesh, before marriage people live along with parents and don’t think about purchasing apartment. The majority of respondents who participated in the survey are between 41 and 50 years (41.5%). The majority of respondents, 144 in total, have a Master’s degree, accounting for 61% of the total respondents. The first occupation class is private service, which has 91 respondents and accounts for 39% of the total respondents, followed by self-employment, which has 73 respondents and accounts for 30.9%, and public service, which has 64 respondents and accounts for 27.1%. The majority of respondents (24.2%), with 57 respondents, fall into the monthly income range of BDT 100,001–150,000, with the second-highest (23.3%), representing 55 respondents, falling into the income range of BDT 50,001–100,000. The majority of respondents (85 out of 236), or 36%, prefer apartment sizes 1001–1500 ft^2^, while the second-highest (84 out of 236) prefer apartment sizes 1501–2000 ft^2^. According to the survey, the highest number of 91 respondents (38.6%) who participated in the survey have an apartment purchase budget of BDT 50,00,001–100,00,000. Hence, it can be said that the demographic profiles and general information of the respondents collected are logical and suitable for this study context.

**Table 1:** Respondents' biographical information.

Variables	Population characteristics	Frequency	Percent
Gender	Male	203	86.0
	Female	33	14.0
Marital Status	Single	5	2.1
	Married	231	97.9
Age	21–30 years	8	3.4
	31–40 years	63	26.7
	41–50 years	98	41.5
	51–60 years	54	22.9
	61 – Above	13	5.5
Education	Diploma	1	.4
	Pass Course	20	8.5
	Bachelor/B. Sc.	46	19.5
	Master	144	61.0
	PhD	25	10.6
Occupation	Public Service	64	27.1
	Private Service	92	39.0
	Self Employed	73	30.9
	Retired	2	.8
	Other	5	2.1
Income (in BDT)	Below 50,000	5	2.1
	50,001–100,000	55	23.3
	100,001–150,000	57	24.2
	150,001–200,000	26	11.0
	200,001–250,000	20	8.5
	250,001–300,000	27	11.4
	Above 300,000	44	18.6
Apartment Size (ft^2^)	Below 1000	4	1.7
	1001–1500	85	36.0
	1501–2000	84	35.6
	2001–2500	38	16.1
	Above 2500	25	10.6
Budget (in BDT)	Below 50,00,000	17	7.2
	50,00,001–100,00,000	91	38.6
	100,00,001–150,00,000	55	23.3
	150,00,001–200,00,000	26	11.0
	200,00,001–250,00,000	14	5.9
	250,00,001–300,00,000	16	6.8
	Above 300,00,000	17	7.2
	Total	236	100.0

*Note:* BDT = Bangladeshi Taka. 1 USD = 85 BDT.

### 4.2 Results of EFA

This study used 63 items to analyze the EFA. The results of KMO and Bartlett’s of Sphericity are as follows. The Kaiser-Meyer-Olkin Measure of Sampling Adequacy is 0.841 and Bartlett’s Test of Sphericity shows significant with the p-value of 0.000 and Chi-Square 10,037.775. This suggests that the data is suitable for conducting the EFA study. Our initial questionnaire had 63 questions. After EFA, we reduced the questions or measurement items to 35, considering the factor loadings. Loadings are the correlations that exist between the resulting components and the initial variables. Cronbach’s alpha coefficient was used to assess the items' internal reliability or consistency. Table 2 shows that the items had an Alpha value greater than 0.7, indicating that the variables are reliable based on the measurement items chosen in EFA. In terms of loadings, the majority of the items have loadings greater than 0.70, which is recommended in the literature (Hair et al., 2016). However, items loadings below 0.60 have been deleted.

**Table 2:** Result of EFA.

New Constructs	Initial items	Revised items	Loadings	Reliability (Cronbach alpha)
Attitude	AT1	AT1	0.923	0.895
	AT2	AT2	0.726	
	AT3	AT3	0.685	
	AT4	AT4	0.924	
	AT5	AT5	0.845	
Subjective norm (SN)	SN1	SN1	0.613	0.798
	SN2	SN2	0.772	
	SN3	SN3	0.696	
	SN4	SN4	0.678	
Perceived behavioural control (PB)	PB1	PB1	0.769	0.862
	PB2	PB2	0.736	
	PB3	PB3	0.846	
	PB4	PB4	0.861	
Perceived physical quality (PPQ)	PF1	PPQ 1	0.688	0.903
	EQ1	PPQ 2	0.653	
	EQ4	PPQ 3	0.616	
	LF1	PPQ 4	0.761	
	PQ2	PPQ 5	0.77	
	LF3	PPQ 6	0.741	
	PQ5	PPQ 7	0.765	
	PQ6	PPQ 8	0.673	
Access to money (AM)	FN1	AM1	0.8	0.916
	FN2	AM2	0.823	
	FN3	AM3	0.817	
	FN4	AM4	0.836	
Favourable government policy (GP)	GI1	GP1	0.793	0.906
	GI2	GP2	0.796	
	GI3	GP3	0.748	
	GI4	GP4	0.708	
	GI5	GP5	0.845	
Purchase intention (PI)	PI1	PI1	0.659	0.864
	PI2	PI2	0.667	
	PI3	PI3	0.66	
	PI4	PI4	0.811	
	PI5	PI5	0.724	

### 4.3 Proposed measurements

The variables and items associated with the extended TPB model are presented in this section. Table 3 lists the variables, as well as their associated items and sources. Appendix A contains the complete set of questions. The first section consists of measuring attitudes. It includes five items that assess respondents' beliefs in various aspects of attitudes such as (i) beneficial, (ii) pleasant, (iii) good, (iv) valuable, and (v) enjoyable. All of the items were derived from the secondary sources listed in Table 3. This questionnaire collected responses from interested buyers looking for apartments in Dhaka using a five-point Likert scale (1 = strongly disagree, 2 = disagree, 3 = neither agree nor disagree, 4 = agree, and 5 = strongly agree). According to the literature, the five-point scale appears to be less confusing and increases the response rate (Babakus and Mangold, 1992).

**Table 3:** Measurement items.

Constructs	Items	Sources
Attitudes	Beneficial Pleasant Good Valuable Enjoyable	Ajzen (1988), AL-Nahdi et al., 2015a, AL-Nahdi et al., 2015b, Al-Nahdi et al., 2015c, AL-Nahdi et al., 2015d, Kamal and Pramanik, 2015a, Kamal and Pramanik, 2015b and Yadav et al. (2018).
Subjective Norm	Guardian influence Spouse’s influence Family members' influence Friend’s influence	Numraktrakul et al. (2012), Chowdhury (2013), AL-Nahdi, (2015), Wibawa et al. (2017) and Judge et al. (2019).
Perceived Behavioural Control	Opportunity Capacity Control Knowledge	AL-Nahdi (2015), Wibawa et al. (2017), Judge et al. (2019) and Khoo et al. (2019).
Perceived Physical Quality	Car parking Air pollution Sound pollution Preferred location Ventilation Design Quality materials Accessibility	Kamal and Pramanik, 2015a, Kamal and Pramanik, 2015b, Chia et al. (2016), Kumar and Khandelwal (2018), Rachmawati et al. (2019) and Sonia (2020).
Access to Money	Enough money Access to home loan Access to borrowings	Islam and Zahur (2016), Jamil et al. (2018), Khoo et al. (2019), Rachmawati et al. (2019) and Sonia (2020).
Favourable Government Policy	Government incentive Policy of affordable home Regulation pressure Special credit Reduction of property transfer fees	Chowdhury (2013), Matisoff et al. (2016), Glaeser et al. (2017) and Alam (2018).
Intention	Commitment to buy Have the recommendation to buy Loyalty to buy Plan to buy Try to buy	AL-Nahdi (2015), Kamal and Pramanik, 2015a, Kamal and Pramanik, 2015b, Kamal et al. (2016), Judge et al. (2019) and Khoo et al. (2019).

The issue of subjective norms was addressed in the second part, which included 5 items adopted from prior studies, as shown in Table 3. The objective of these items was to measure the influences of purchase intention. The five items are (i) parent’s influence, (ii) child’s influence, (iii) spouse’s influence, (iv) friend’s influence. Respondents were asked to rate their level of agreement on a five-point scale ranging from strongly disagree to strongly agree.

Third, the issue of perceived behavioural control was addressed. It was based on the previous studies listed in Table 3. The goal of these items was to assess the impact on purchasing intent. The five items are (i) opportunity (easy market access) in deciding to buy an apartment, (ii) capacity to make a buying decision, (iii) control to buy an apartment, and (iv) knowledge about the apartment to make a buying decision. Respondents were asked to rate their level of agreement on a five-point scale ranging from strongly disagree to strongly agree.

The fourth section included a measurement of perceived physical. This section measured the following eight items: (i) parking, (ii) air pollution, (iii) sound pollution, (iv) preferred location, (v) ventilation, (vi) functional design, (vii) quality building materials (viii) convenient accessibility. Respondents were asked to rate their level of agreement on a five-point scale ranging from strongly disagree to strongly agree.

The fifth independent variable, access to money, was determined by asking buyers whether they (i) having enough money, (ii) having access to a home loan, (iii) can borrow money from relatives, (iv) can borrow money from friends. Respondents were asked to rate their level of agreement on a five-point scale that ranged from strongly disagree to strongly agree.

The sixth independent variable, favourable government policy, was assessed by asking whether buyers are influenced in their buying decisions by (i) government incentives, (ii) affordable housing policies, (iii) regulatory pressure, (iv) special credit, and (v) property transfer fee reductions.

The seventh component deals with purchase intention, which is followed by customer behaviour. The respondents were asked whether they (i) had made a promise to buy, (ii) if they had gotten any recommendations to buy, (iii) if they were loyal to any company, (iv) if they had any plans to buy, and (v) if they would try to acquire an apartment. They were asked to rate their level of agreement on a five-point scale that ranged from strongly disagree to strongly agree.

### 4.4 Testing the propositions

**Figure 2:**
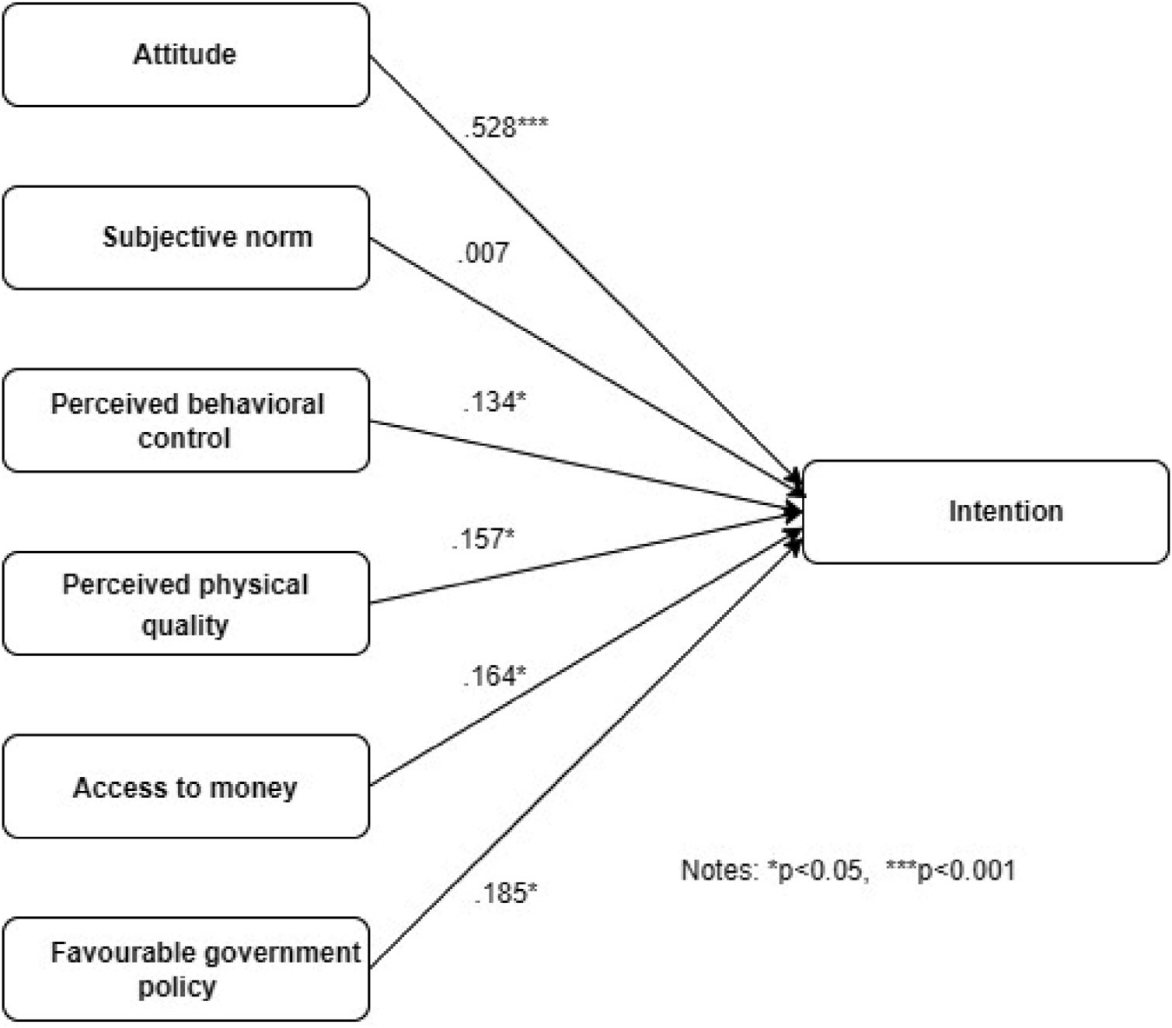
Graphical representation of the causal relationships.

We also tested the proposition using multiple regression method. The summary reports are presented in Table 4 and also graphically in Figure 2. The model summary of SPSS output reports that R square values is .484 indicating 48 percent of the variances is explained by the predictors. The result of ANOVA reveals that the F value is 35.769 with p value 0.000 indicating the overall model relationship is significant. Regarding the individual propositions the findings reveal that all the relationships are significant except P2. The predictors namely, attitude, perceived behavioural control, perceived physical quality, access to money and the government favourable policy towards apartments buying have a significant positive relation with the intention. However, there is no sufficient statistical evidence to support the P2. The reason might be cultural change took place in Bangladesh, especially, in urban areas (Kamal et al., 2016). As such, people in urban areas rarely bother about the opinion of guardian, spouse, family member, friends, and reference group in buying apartment.

**Table 4:** Result of multiple regression.

Propositions	Relationships	Std. Beta	Std. Error	t value	P value	Decision
P1	Attitude → Intention to purchase	.528	.055	9.553	.000	Supported
P2	Subjective Norm → Intention to purchase	.007	.058	.089	.284	Not supported
P3	Perceived behavioral control → Intention to purchase	.134	.049	2.494	.013	Supported
P4	Perceived physical quality → Intention to purchase	.157	.081	2.365	.025	Supported
P5	Access to money → Intention to purchase	.164	.077	2.126	.017	Supported
P6	Govt. policy ⇒ Intention to purchase	.185	.043	1.982	.025	Supported

## 5 Theoretical and practical implications

The present research used an extended TPB model to examine consumers' apartment buying behaviour in Dhaka. Aside from TPB, the findings indicated numerous new characteristics that can be used to predict behavioural intention, including perceived physical quality, access to money, and favourable government policy. The physical quality of service leads to satisfaction, and contentment leads to intention, according to the service quality model (Priporas et al., 2017; Parasuraman et al., 1988). The high cost of apartments in Bangladesh has been discovered to be a barrier to buyers (Barua et al., 2010). As a result, having access to money could potentially enhance the desire to buy, particularly in Bangladesh. Furthermore, it is believed that favourable government policies are necessary to encourage customers to purchase real estate (Rahman, 2021). The findings of this study show that buying an apartment is helpful and valuable to consumers and that the demand of family and friends also contributes to the purchase of an apartment that meets the factor-subjective standard. It also recognised the characteristics that influence buying behaviour, such as the ability, capacity, and control over a purchase. The interaction of the apartment with the environment and its interior amenities, on the other hand, influences the decision to purchase an apartment with money available through a loan or other means. This study found that government initiatives such as incentives and regulatory measures that encourage people to own apartments play an important role. The TPB was employed to create initial factors including subjective norm, attitude, and perceived behavioural control in this study. Using exploratory factor analysis (EFA), the researchers discovered three additional important components. Additional aspects include physical quality perceptions, financial access, and favourable government policies. Therefore, this study extended the TPB by integrating three additional contextual factors for studying behavioural intention. The study adds to the body of knowledge by highlighting how money, government policy, and perceived physical quality affect apartment purchasing. The expanded TPB model, rather than the original TPB model, appears to be a better fit for the circumstance. By establishing a model for understanding behavioural intention, this study contributes significantly to the literature.

This study also bring practical implications. First, the proposed model helps practitioners to study the perceptions of consumers in real estate industry with an aim to understand their purchase intention. Second, the empirical evidences of this study also provide useful information to the practitioners engaged in the real estate industry for better decision making regarding designing their products and services.

## 6 Conclusion

It is critical for both buyers and the industry to understand the elements that influence apartment purchases in real estate markets. To succeed in the real estate industry, companies must first comprehend consumer behaviour while purchasing apartments. The goal of the study is to create a model for analysing behavioural intentions to purchase homes in Bangladesh. TPB was used as an underlying theoretical framework. This study identified other relevant factors by conducting exploratory factor analysis (EFA). Finally, a new proposal is presented.

Based on the extended TPB, this study created a theoretical model of behavioural intention to investigate factors influencing apartment buying. This paper adds to the discussion by presenting three additional factors in addition to the one proposed by TPB. The addition of three variables (perceived physical quality, access to money, and favourable government policy) is expected to improve the existing theory’s explanatory power in the given context.

Several limitations apply to this study. First, this study investigated the relevant factors using EFA without delving into the causal relationship between the independent and dependent variables. Second, no moderator or mediator was considered in this study. Therefore, future research could include a mediator and moderator. Although gender and age are commonly used as moderators, it would be interesting to see access to money in this model as either a moderator or a mediator. Finally, this study only takes into account the effects of psychological factors on consumers' behavioural intentions. Other factors, such as migration to Dhaka and capital pull factors, are not taken into account. As a result, future research could delve deeper into the migration and pull factors.

## Declarations

### Author contribution statement

Muhammad Ariful Islam, Zainil Hanim Saidin, Meor Azli Ayub, Md Shamimul Islam: Conceived and designed the experiments; Analyzed and interpreted the data; Contributed reagents, materials, analysis tools or data; Wrote the paper.

### Funding statement

This research did not receive any specific grant from funding agencies in the public, commercial, or not-for-profit sectors.

### Data availability statement

The authors do not have permission to share data.

### Declaration of interests statement

The authors declare no conflict of interest.

### Additional information

No additional information is available for this paper.

## Figures and Tables

**Table:** 

**Gender:** 1. Male 2. Female	**Marital Status** 1. Single 2. Married
**Family Size:** 1. One 2. Two 3. Three 4. Four 5. More _____________	**Age:** 1. 21–30 years 2. 31–40 years 3. 41–50 years 4. 51–60 years 5. 61–Above
**Education:** 1. Diploma 2. Pass Course 3. Bachelor/B. Sc. 4. Master 5. PhD 6. Other _____________	**Occupation:** 1. Public Service 2. Private Service 3. Business/Self Employed 4. Retired 5. Other _____________
**Monthly Family Income (BDT):** 1. Below 50,000 2. 50,001–100,000 3. 100,001–150,000 4. 150,001–200,000 5. 200,001–250,000 6. 250,001–300,000 7. Above 300,000	**Preferred Apartment Size (Sft):** 1. Below 1000 2. 1001–1500 3. 1501–2000 4. 2001–2500 5. Above 2500
**Budget for Apartment (BDT):** 1. Below 50,00,000 2. 50,00,001–100,00,000 3. 100,00,001–150,00,000 4. 150,00,001–200,00,000 5. 200,00,001–250,00,000 6. 250,00,001–300,00,000 7. Above 300,00,000	

**Table:** 

1.0	Attitudes [to what extent you agree with the following statement to buy an apartment from the real estate market in Dhaka City]					
		**Strongly Disagree**	**Disagree**	**Neither Agree nor Disagree**	**Agree**	**Strongly Agree**
**1.1**	Buying an apartment is beneficial for me	1	2	3	4	5
**1.2**	Buying an apartment is pleasant for me	1	2	3	4	5
**1.3**	Buying an apartment is good for me	1	2	3	4	5
**1.4**	Buying an apartment is valuable for me	1	2	3	4	5
**1.5**	Buying an apartment is enjoyable for me	1	2	3	4	5
2.0	Subjective Norm [to what extent you rate the following attributes]					
**2.1**	My guardians encourage me to have my own house	1	2	3	4	5
**2.2**	My spouse encourages me to own an apartment	1	2	3	4	5
**2.3**	My family members expect me to own an apartment	1	2	3	4	5
**2.4**	My friends influence me to buy an apartment	1	2	3	4	5
3.0	Perceived Behavioural Control [to what extent you believe that you have control on following attributes]					
**3.1**	I have enough opportunity in making purchase decision	1	2	3	4	5
**3.2**	I have the capacity to make a purchase decision	1	2	3	4	5
**3.3**	I have enough control in my purchase decision	1	2	3	4	5
**3.4**	I have enough knowledge to make purchase decision	1	2	3	4	5
4.0	Perceived Physical Quality [to what extent you believe that your apartment has the following characteristics]					
**4.1**	I prefer to buy an apartment that have a convenient parking space	1	2	3	4	5
**4.2**	I prefer to buy an apartment that is free from air pollution	1	2	3	4	5
**4.3**	I prefer to buy an apartment that is free from sound pollution	1	2	3	4	5
**4.4**	I prefer to buy an apartment that is built in my preferred location	1	2	3	4	5
**4.5**	I prefer to buy an apartment that has sufficient ventilation	1	2	3	4	5
**4.6**	I prefer to buy an apartment that has a functional design	1	2	3	4	5
**4.7**	I prefer to buy an apartment that used good quality building materials	1	2	3	4	5
**4.8**	I prefer to buy an apartment that has a convenient accessibility	1	2	3	4	5
5.0	Access to Money [to what extent you believe that you have access to the following financial facilities in buying an apartment]					
**5.1**	I have sufficient money at hand to buy an apartment	1	2	3	4	5
**5.2**	I have access to home loan facility	1	2	3	4	5
**5.3**	I can borrow money from my relatives if I want	1	2	3	4	5
**5.4**	I can borrow money from my friends if I want	1	2	3	4	5
6.0	Government Incentives, Policies and Regulation [to what extent you believe that government policy will influence you buying an apartment]					
**6.1**	Government incentive has impact on my purchase decision	1	2	3	4	5
**6.2**	Mandatory policy of affordable option in large housing projects can help me to purchase	1	2	3	4	5
**6.3**	Regulatory pressure can influence me to purchase home in Dhaka city	1	2	3	4	5
**6.4**	Special credit facilities from Government can influence me to purchase apartment	1	2	3	4	5
**6.5**	Reduction of property transfer fees from Government can influence me to purchase	1	2	3	4	5
7.0	Purchase Intention [to what extent you believe you will buy an apartment]					
7.1	I have commitment to buy	1	2	3	4	5
7.2	I got recommendation to buy	1	2	3	4	5
7.3	I have loyalty to buy an apartment	1	2	3	4	5
7.4	I have plan to buy apartment	1	2	3	4	5
7.5	I will try to buy an apartment	1	2	3	4	5

## Appendix A

Sample of Questionnaire after EFA.

1. Demographic Information:

Following items measure the perceptions regarding variables of interest.
